# Alternative-Dose versus Standard-Dose Trivalent Influenza Vaccines for Immunocompromised Patients: A Meta-Analysis of Randomised Control Trials

**DOI:** 10.3390/jcm8050590

**Published:** 2019-04-29

**Authors:** Jiun-Ji Lai, Chin Lin, Ching-Liang Ho, Po-Huang Chen, Cho-Hao Lee

**Affiliations:** 1 Division of Infectious Diseases and Tropical Medicine, Department of Internal Medicine, Tri-Service General Hospital, National Defense Medical Center, Taipei 11490, Taiwan; s493011074@yahoo.com.tw; 2 School of Public Health, National Defense Medical Center, Taipei 11490, Taiwan; xup6fup0629@gmail.com; 3 Department of Research and Development, National Defense Medical Center, Taipei 11490, Taiwan; 4 Division of Hematology and Oncology Medicine, Department of Internal Medicine, Tri-Service General Hospital, National Defense Medical Center, Taipei 11490, Taiwan; charileho22623@gmail.com

**Keywords:** influenza vaccine, immunocompromised, cancer, chemotherapy, transplant, booster dose, double dose, high dose, trivalent, meta-analysis

## Abstract

The study compared immunogenicity and safety between alternative higher-dose and standard-dose trivalent vaccines in immunocompromised individuals. A literature search was performed using the PubMed, Embase, and Cochrane databases from inception until March 2019 to identify studies comparing the immunogenicity of alternative higher-dose (including high-dose, double-dose, and booster-dose vaccines) and standard-dose trivalent influenza vaccines in patients who underwent transplantation or chemotherapy. Effect estimates from the individual studies were derived and calculated using the DerSimonian and Laird random-effect model. The protocol for this systematic review is registered with PROSPERO (number CRD42019129220). Eight relevant studies involving 1020 patients were included in the systematic review and meta-analysis. The meta-analysis demonstrated that the higher-dose strategy provided had significantly superior seroconversion and seroprotection for A/H1N1 strains than the standard dose. Regarding H3N2 and B strains, no differences in immunogenicity responses were noted. No differences in safety were observed between the vaccination strategies. Alternative higher-dose vaccination strategies appear to associate with superior immunogenicity responses for A/H1N1 strains, and the strategies were generally well tolerated in immunocompromised populations. Future studies should clarify the optimal timing, frequency and dose of vaccination and assess whether these strategies improve vaccine immunogenicity and clinical outcomes.

## 1. Introduction

Influenza is associated with substantial increases in morbidity and mortality each year. Globally, these annual epidemics are estimated to result in approximately 3–5 million cases of severe illness and approximately 290,000–650,000 deaths [1]. Immunocompromised individuals, including those with solid organ transplants, haematopoietic stem cell transplants, solid cancers, or haematologic malignancy, are at high risk of influenza-associated complications such as severe bacterial pneumonia, intensive care admission, a need for mechanical ventilation, or death [2]. In addition to the direct effects of influenza infection, the disease has also been associated with allograft dysfunction as well as acute and chronic rejection in patients who previously underwent transplantation [3]. Thus, various expert guidelines recommend inactivated trivalent influenza vaccination for immunocompromised persons [4], and it is the most effective strategy for reducing the incidence of influenza.

Several different types of vaccines have been licensed, but only inactivated formulations are suitable for immunocompromised individuals [5]. For decades, the standard influenza vaccine was an un-adjuvanted trivalent inactivated formulation containing two A strains and one B strain. The specific strains included in the vaccine change annually based on the recommendation of the WHO (World Health Organization).

Though annual immunization with inactivated influenza vaccine has been recommended, the response to influenza vaccination in immunocompromised patients is heterogeneous and suboptimal, with strain-specific rates of seroprotection that range from 15 to 90% [6,7,8], generally lower than the response in immunocompetent individuals [9]. Therefore, various strategies to improve influenza vaccine immunogenicity responses have been attempted including two vaccine doses (booster) in the same influenza season [10] and high-dose vaccines (two- or four-fold dose) [7,11,12,13,14,15,16]; in a similar vein, these alternative ‘higher-dose’ influenza vaccination strategies have been developed to ameliorate serologic responses. The increase in serum antibody responses is expected to be correlated with an increase in vaccine immunogenicity responses [17].

However, there are limited data concerning the immunogenicity responses of higher-dose influenza vaccines in immunocompromised populations, and there is no consensus regarding whether the higher-dose strategy provides superior immunogenicity and safety than the standard formulation in these subjects, reflecting a need to make evidence-based decisions to shift clinical practice to ensure the routine use of the most effective vaccines in immunogenicity responses. Therefore, we performed a meta-analysis of randomized controlled trials (RCTs) to compare the immunogenicity and safety of alternative higher-dose and standard-dose trivalent influenza vaccines in immunocompromised subjects, including patients who underwent organ or haematopoietic stem cell transplantation and those with solid tumors or haematologic malignancy who are receiving chemotherapy.

## 2. Materials and Methods

### 2.1. Data Sources and Searches

We performed a systematic literature search using electronic datasets (i.e., PubMed, Embase, Cochrane Central, and Web of Science databases) for RCTs that assessed the immunogenicity and safety of alternative higher-dose influenza vaccines in immunocompromised patients. A manual screening for references from original articles, previous systematic reviews, and conference abstracts was also performed to identify eligible trials up to 16 March 2019. We followed the Preferred Reporting Items for Systematic Reviews and Meta Analyses guidelines for performing the systematic reviews and meta-analyses of RCTs [18]. The protocol for this systematic review is registered with PROSPERO (number CRD42019129220).

### 2.2. Eligibility Criteria

Studies involving immunocompromised patients (patients with solid tumors or haematologic malignancy who are undergoing chemotherapy or post-transplantation patients, no age limitations) in which the intervention and comparator consisted of a higher-dose trivalent influenza vaccine (>15 mcg haemagglutinin antigen per viral strain) and a standard-dose influenza vaccine, respectively, were included.

We included studies that reported immunogenicity and safety outcomes. The primary outcome was immunogenicity, including seroconversion and seroprotection rates. The seroconversion rate was calculated as the percentage of subjects with either a pre-vaccination titre < 10 and a post-vaccination titre ≥ 40, or, alternatively a four-fold increase in titres versus a pre-vaccination titre ≥ 10. The seroprotection rate was defined as the percentage of subjects with a post-vaccination titre ≥ 40 for each strain. Secondary outcomes were adverse events. Adverse events were graded using Common Terminology Criteria for Adverse Events (CTCAE) [19] as mild (no interference in daily activities, CTCAE grade 1), moderate (some interference in daily activities, CTCAE grade 2), and severe (unable to participate in daily activities, CTCAE grade 3). Serious adverse events (SAEs) were considered life-threatening or medically important events resulting in disability or hospitalization. Peer-reviewed RCTs were included without language limitation.

We excluded studies that used pandemic, avian, or swine influenza vaccines, as well as those that used monovalent or bivalent seasonal influenza vaccines. Trials identified from the literature search were initially screened for relevance based on titles and abstracts. Studies that did not meet these criteria during the title and abstract screen were excluded. Full-text reviews were then performed using the studies included after screening to ensure that they met the eligible criteria.

### 2.3. Data Extraction and Quality Assessment

Two reviewers (Cho-Hao Lee and Po-Huang Chen) appraised all eligible citations independently and extracted various data from original trial reports, including author names, publication year, geographic regions, trial registration number, study designs, sample size and participant characteristics (e.g., mean age, inclusion criteria, vaccine). The outcomes of interest including immunogenicity (seroconversion and seroprotection rates) and safety (the number of patients with SAEs) were also extracted. For dichotomous outcomes of immunogenicity (seroconversion and seroprotection rates), we extracted the proportion rate of both the experimental and comparator arms. For dichotomous outcomes of safety, we extracted the number of people with the event per arm. CHL double-checked the data to minify the possible typing or entry error and limit selection bias. To address the risk of bias with multiple data extractors, standardized templates were created. The quality of trials was appraised using the *Cochrane Handbook for Systematic Reviews of Interventions* [20]. Seven domains—namely selection, attrition, performance, detection, reporting, contamination, and other types of bias—are listed in the Figure S1. Any disagreement between the two independent reviewers was resolved via group discussions [21]. Risk of bias graphs were generated using Review Manager 5.3 software [22].

### 2.4. Data Synthesis and Analysis

Data analysis was conducted as recommended in the *Cochrane Handbook for Systematic Reviews of Interventions* [23]. We used both random- and fixed-effects modelling to pool all outcomes and interpreted random-effects meta-analyses with consideration of the complete distribution of effects. All meta-analyses were conducted using the DerSimonian and Laird random-effect model [24]. The Mantel-Haenszel method (fixed-effects model) [25] was also described. We calculated dichotomous outcomes for immunogenicity using the risk difference (RD) and 95% confidence interval (95% CI), and safety outcomes were assessed using the relative risk (RR) and 95% CI.

Heterogeneity and publication biases were evaluated using I2 statistic and funnel plots with Egger’s test [26]. Statistically significant heterogeneity was defined as I2 > 50%. The cause of heterogeneity was investigated for main outcomes using sensitivity tests and a mixed-effects meta-regression model with variables including mean age and underlying status (under chemotherapy or post-transplant) [23]. All statistical analyses were performed using the ‘metafor’ and ‘meta’ [27,28] packages of R software version 3.3.1 [29]. A two-tailed significance test (*p* = 0.05) denoted statistical significance without multiplicity correction in all exploratory analyses.

### 2.5. Subgroup Analyses

Subgroup analyses were performed to detect clinical heterogeneities. We separated the group by vaccine dose (double-dose (total, 30 mcg) and high-dose (total, 60 mcg)), patient age (children (mean age < 18 years), and adult (mean age ≥ 18 years)) and causes of immunocompromise (malignancy requiring chemotherapy and transplantation).

## 3. Results

### 3.1. Characteristics of the Identified Studies

**Figure 1:**
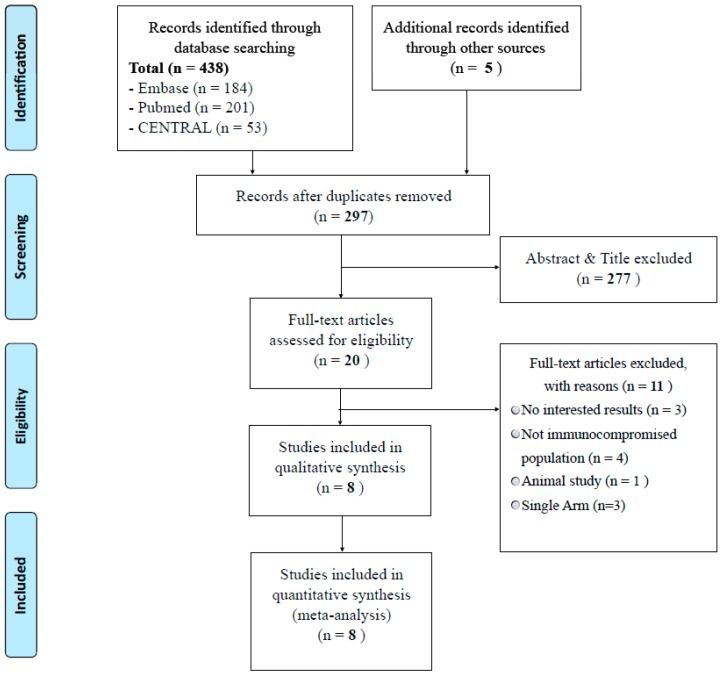
Preferred reporting items for systematic reviews and meta analyses flowchart of study selection.

In total, 443 articles were identified in the initial literature search, and full-text reviews were conducted for 20 articles after abstract and title screening. Finally, eight articles [7,10,11,12,13,14,15,16] involving 1020 patients were included in the systematic review and meta-analysis (Figure 1).

Basic characteristics are listed in Table 1. Of the included articles, five trials were double-blind [7,11,13,14,15], and the others had an open-label design [10,12,16]. The present meta-analysis contained three phase I [11,13,15], three phase II [12,14,16] and two phase III trials [7,10] with relative good quality (Table 1 and Figure S1). The dominate influenza strains were A/H3N2 and A/H1N1 among the studies. Five studies focused on transplant recipients [7,10,11,13,16], and three studies examined patients who received induction or maintenance chemotherapy [12,14,15].

### 3.2. Influenza Vaccine Characteristics and Vaccination Strategy

The alternative higher-dose influenza vaccination utilized in our meta-analysis had three different strategies.

Six trials [7,11,12,13,14,15] used a single injection of a high-dose inactivated trivalent influenza vaccine (HD-IIV3; Fluzone^®^ High-Dose, Sanofi Pasteur, Swiftwater, PA, USA), which contains four-fold more antigen than the standard-dose inactivated trivalent influenza vaccine (SD-IIV3). HD-IIV3 contains 60 mcg each of three antigens (H1N1, H3N2 and influenza B) instead of the usual 15 mcg per antigen in SD-IIV3.

One trial [16] used a double-dose simultaneous intramuscular injection of a Mutagrip standard dose (15 mcg of trivalent split-inactivated haemagglutinin antigen per strain, Sanofi Pasteur, France), and one trial [10] used booster injections of a Mutagrip standard dose (15 mcg of haemagglutinin antigen per strain per strain, Sanofi-Pasteur MSD). In the booster group, patients received the standard intramuscular single-dose vaccination and a second strain of the same vaccine after 5 weeks.

The comparator group in all trials received SD-IIV3 containing 15 mcg of antigen each for two A strains (H1N1 and H3N2) and one B strain. The composition of influenza vaccines depended on the annual recommendation of the WHO Global Influenza Surveillance and Response System.

### 3.3. Vaccination Immunogenicity in the Identified Studies

All eight studies evaluated immunogenicity by measuring pre-vaccination and post-vaccination strain-specific influenza antigen titres, as well as seroconversion and seroprotection rates.

GiaQuinta et al. [11] randomized 38 pediatric subjects to receive either HD-IIV3 or SD-IIV3. A higher proportion of the HD-IIV3 group developed seroconversion for A/H3N2 than in the SD group (54% vs. 13%; *p* = 0.011), but the seroprotection rates for A/H1N1 (95% vs. 80%; *p* = 0.14) and influenza B (46% vs. 47%; *p* = 0.94) did not significantly differ.

McManus et al. [15] reported 50 pediatric patients with acute lymphoblastic leukemia who had received maintenance chemotherapy for at least four weeks after their first remission. Immunogenicity data, including seroconversion and seroprotection rates, were similar between the HD-IIV3 and SD-IIV3 groups for all three strains.

In a study by Hakim et al. [12], 44 children with leukemia or solid tumors were enrolled. Among participants with leukemia, there were no significant differences in seroconversion or seroprotection rates between patients who received HD-IIV3 or SD-IIV3 except that slightly more participants who received HD-IIV3 achieved seroprotection against A/H3N2 and B antigens (A/H3N2: 80% vs. 67%, B: 91% vs. 83%). No differences in seroconversion and seroprotection were observed between the HD-IIV3 and SD-IIV3 groups among participants with solid tumours.

In a study by Halasa et al. [13], which enrolled 44 adult stem cell haematopoietic transplant recipients, a trend of higher seroconversion rates was noted for A/H3N2 in the HD-IIV3 group than in the SD-IIV3 group (81% vs. 36%, *p* = 0.004); however, there were no differences for the A/H1N1 and B strains regarding seroconversion and seroprotection.

Jamshed et al. [14] reported significantly improved seroconversion rates for HD-IIV3 for all three strains (RD (HD-IIV3 minus SD-IIV3) = 26% for A/H1N1, 22% for A/H3N2 and 26% for B) among 105 adults with malignancy who were receiving chemotherapy. Seroprotection rates were similar between HD-IIV3 and SD-IIV3 for all three strains (96% vs. 90% for A/H1N1, 96% vs. 96% for A/H3N2, and 88% vs. 72% for B).

Natori et al. [7] analyzed 161 adult solid organ transplant recipients who were randomized to receive either HD-IIV3 or SD-IIV3. Rates of seroconversion for the A/H1N1, A/H3N2, and B strains were 40.5%, 57.1%, and 58.3%, respectively, for HD-IIV3, versus 20.5%, 32.5% and 41.6%, respectively, for SD-IIV3 (*p* = 0.006, 0.002 and 0.028, respectively). The rates of seroconversion for at least one of the three influenza antigens were 78.6% and 55.8% for the HD-IIV3 and SD-IIV3 vaccines, respectively (*p* = 0.002). Seroprotection rates ranged 74–94%, and no significant difference was observed between the vaccine strategies.

### 3.4. Double Simultaneous Dose Influenza Vaccine

Mombelli et al. [16] randomized 79 adult solid organ transplant recipients to receive a double-dose inactivated trivalent influenza vaccine (DD-IIV3) or SD-IIV3. There was a non-significant trend for higher probability of seroconversion for all three viral strains among patients in the DD-IIV3 group. Meanwhile, DD-IIV3 was linked to a higher probability of seroconversion to all three strains (87.5% vs. 69.2% at four weeks after vaccination, *p* = 0.048).

### 3.5. Booster-Dose Influenza Vaccine

Cordero et al. [10] reported the results of the TRANSGRIPE 1–2 study, which evaluated the efficacy and safety of a booster-dose influenza vaccine in 499 solid organ transplant recipients. Using a modified intention-to-treat analysis, the seroconversion rate was higher in the booster group than in the SD-IIV3 group at 10 weeks for A/H1N1 (46.7% vs. 32.7%, *p* = 0.046), but not for the other influenza strains. The seroprotection rate was higher in the booster group than in the control group for all three strains (A/H1N1: 54% vs. 43.2%, *p* = 0.026; A/H3N2: 56.9% vs. 45.5%, *p* = 0.020; influenza B: 83.4% vs. 71.8%, *p* = 0.004).

### 3.6. Meta-Analysis of Vaccine Efficacy

#### 3.6.1. Immunogenicity

**Figure 2:**
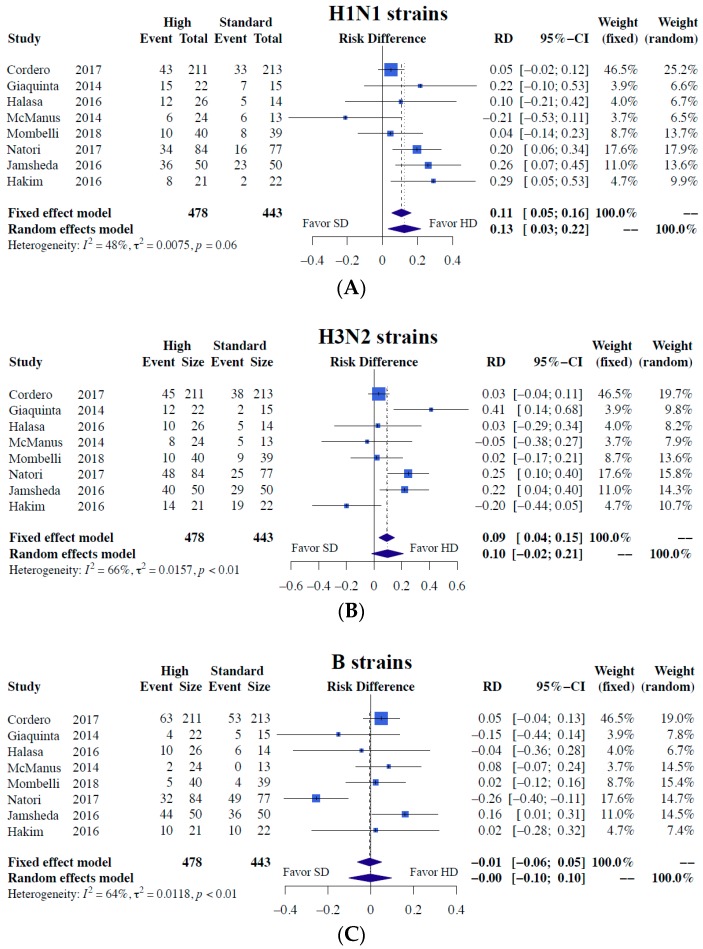
Meta-analyses of seroconversion differences between alternative higher-dose and standard-dose trivalent influenza vaccines [7,10,11,12,13,14,15,16]. (**A**) H1N1 strains (**B**) H3N2 strains, and (**C**) B strains. Risk difference and 95% confidence intervals were used as measures of immunogenicity responses for dichotomous variables.

**Figure 3:**
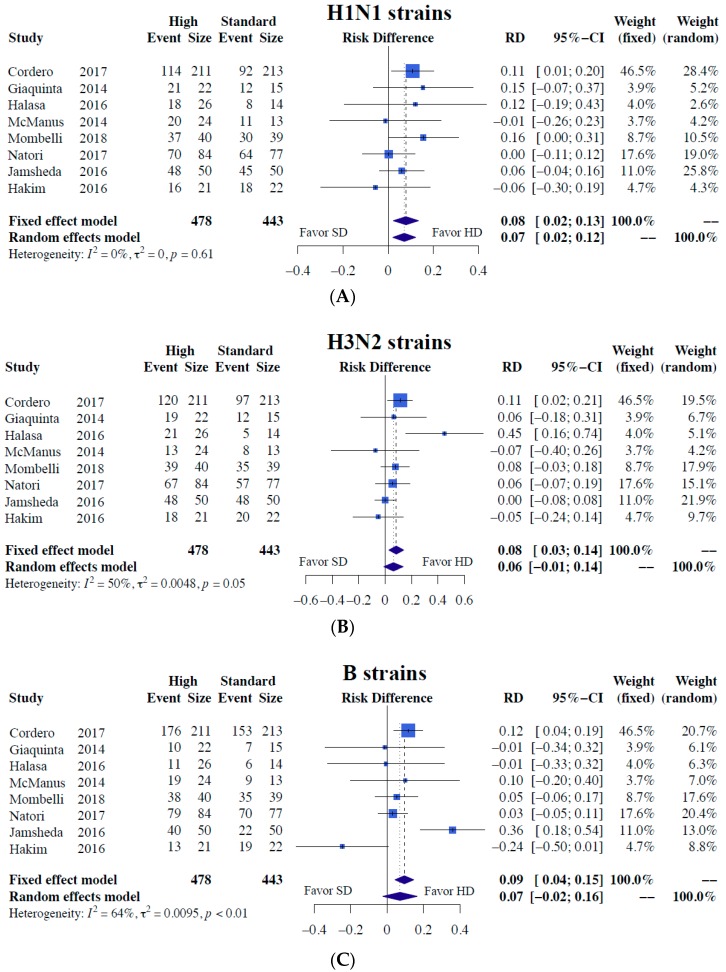
Meta-analyses of seroprotection differences between alternative higher-dose and standard-dose influenza vaccines [7,10,11,12,13,14,15,16]. (**A**) H1N1 strains, (**B**) H3N2 strains, and (**C**) B strains. Risk difference and 95% confidence intervals were used as a measure of immunogenicity responses for dichotomous variables.

Our meta-analysis results demonstrated significantly superior immunogenicity responses against A/H1N1 regarding both seroconversion (RD = 0.13, 95% CI = 0.03–0.22, I2 = 48%) and seroprotection (RD = 0.07, 95% CI = 0.02–0.12, I2 = 0%) for higher-dose vaccines than the standard dose (Figure 2 and Figure 3). In terms of H3N2 strains, the higher-dose strategy was associated with superior seroconversion (RD = 0.10) and seroprotection (RD = 0.06), albeit without significance (Figure 2 and Figure 3). No difference in immunogenicity was noted for the B strain (Figure 2 and Figure 3). Detailed results including all the meta-analysis result of immunogenicity outcomes in risk ratio were provided in (Table S3).

#### 3.6.2. Subgroup Analyses of Immunogenicity

Subgroup analyses were performed to examine several clinical heterogeneities (vaccination dosage, mean age and underlying status; details are described in Table 2).

First, we separated the entire population by influenza vaccine dosage into double-dose (30 mcg) and high-dose (60 mcg) subgroups. Two studies using double-dose vaccination reported significantly superior immunogenicity responses regarding seroprotection for the A/H1N1 (RD = 0.121, 95% CI = 0.040–0.202) and A/H3N2 strains (RD = 0.098, 95% CI = 0.027–0.168). Six studies demonstrated significantly better seroconversion for H1N1 for high-dose vaccination (RD = 0.180, 95% CI = 0.065–0.295).

Second, we separated the entire population by underlying status into two different subgroups—namely patients who underwent transplantation and patients with malignancy requiring chemotherapy. Patients who underwent transplantation experienced significant benefits from higher-dose vaccination regarding both seroconversion and seroprotection for H1N1 and H3N2 strains. Concerning H1N1, significant risk reductions of 9.2 (95% CI = 0.023–0.162, *p* < 0.05) and 8.9% (95% CI = 0.027–0.152; *p* < 0.05) were achieved for seroconversion and seroprotection, respectively. Regarding H3N2 strains, these values were 13.9 (95% CI = 0.002–0.276, *p* < 0.05) and 10.8% (95% CI = 0.027–0.190, *p* < 0.05), respectively.

Third, we separated the entire population by mean patient age (>18 years and <18 years old). Higher-dose influenza vaccination had more favorable effects in patients aged >18 years regarding seroconversion and seroprotection for H1N1 and H3N2 strains.

The pooled numbers needed to vaccinate (NNVs) for HD-IIV3 compared with SD-IIV3 regarding seroconversion and seroprotection were 7.7 and 14.3, respectively, for H1N1 strains. When considering only post-transplant populations, the NNVs for HD-IIV3 compared with SD-IIV3 concerning seroconversion and seroprotection were 10.1 and 11.2, respectively, for H1N1 strains and 7.2 and 9.3, respectively, for H3N2 strains.

### 3.7. Safety

Concerning safety, we collected overall adverse events and separated the events by severity (Table 3). All safety meta-analyses revealed no significant differences between higher-dose and standard-dose vaccination for both overall adverse events (Risk Ratio = 1.021, 95% CI = 0.677–1.540, *p* = 0.921) and SAEs (Risk Ratio = 0.823, 95% CI = 0.551–1.228, *p* = 0.339). The incidence of graft-versus-host rejection did not differ between the groups (RR = 1.379, 95% CI = 0.386–4.933, *p* = 0.621).

## 4. Discussion

The impact of influenza in immunocompromised patients is a serious public health concern, and it is important to evaluate vaccine immunogenicity responses for this population to help inform evidence-based immunization recommendations and programs. This is the first systematic review and meta-analysis of alternative higher-dose trivalent seasonal influenza vaccines that utilized immunogenicity and safety outcomes from RCTs.

The meta-analyses performed in this study found that alternative higher-dose vaccines were more effective in immunogenicity responses than the standard dose vaccine in reinforcing seroconversion and seroprotection for the H1N1 strain in post-transplant and chemotherapy-treated patients. Even when outcomes from booster- and double-dose influenza vaccination trials were excluded, HD-IIV3 was more effective in immunogenicity responses than SD-IIV3 in promoting seroconversion for the H1N1 strain. Regarding post-transplant patients, HD-IIV3 was more effective in terms of seroconversion and seroprotection for both H1N1 and H3N2.

The absolute impact of HD-IIV3 on immunogenicity response is demonstrated through NNV calculations, a metric often used to assess the clinical relevance of vaccines that is akin to the number needed to treat, which is used to assess the impact of other medical interventions. The NNVs of HD-IIV3 instead of SD-IIV3 to achieve seroconversion and seroprotection were 7.7 and 14.3, respectively, based on the pooled absolute RR from the meta-analyses of randomized studies. In addition to immunogenicity, our meta-analysis also highlighted that the higher-dose influenza vaccine was well tolerated in these patients without causing excess harm; indeed, different dosages of the vaccine had a neutral effect on adverse events irrespective of severity. The neutral safety effect was consistent with the findings of previous large RCTs. A pivotal phase III trial in older adults demonstrated that HD-IIV3 (60 μg) had similar safer and greater immunogenicity than SD-IIV3 (15 µg HA/strain) [30]. DiazGranados et al. reported lower SAE rates in the HD-IIV3 group than in the SD-IIV3 group, and the risk of developing at least one SAE during the study was significantly lower in the HD-IIV3 group (RR = 0.92; 95% CI = 0.85–0.99) [31].

A number of reviews [32,33,34] and two meta-analyses [35,36] specifically evaluated the role of vaccines against several illnesses, including influenza, in immunocompromised patients because, compared with healthy adults, the immunogenicity of vaccines may be reduced and the balance between potential benefits and harms of influenza vaccines is difficult to establish. Moreover, in all of these reviews, one of the unresolved issues was the role played by new strategies to improve vaccine response, such as higher doses or adjuvant regimens. Therefore, our results illustrate the need to clarify the usefulness of higher-dose administration in terms of immunogenicity in patients with impaired immune systems.

Since 2003, subjects with weakened immune systems have been deemed at ‘high risk’ of adverse outcomes because of infection by seasonal influenza, as indicated by the WHO [37], following immunization with SD-IIV3. Recently, RCTs [38,39] and meta-analyses [40] found that HD-IIV3 was more effective regarding seasonal influenza immunogenicity and clinical outcomes for elderly or nursing home patients. The Food and Drug Administration of US also approved the administration of HD-IIV3 for subjected aged >65 years for influenza prevention in 2017.

Older populations have weakened immune systems; thus, it is reasonable that HD-IIV3 may provide similar vaccine efficacy in immunocompromised patients. Although no studies have examined the efficacy of HD-IIV3 in immunosuppressed populations, some clinical trials suggested that HD-IIV3 is more immunogenic than SD-IIV3 [7,11,13,14,15].

Thus, based on the results of our meta-analysis, we highlighted the broad impact of influenza vaccines in subjects with impaired immune systems and the possibility that HD-IIV3 provides better immunogenicity, including seroconversion and seroprotection, for the H1N1 and H3N2 strains than SD-IIV3.

Furthermore, adverse events do not appear to represent a significant safety issue or obstacle to the acceptability of HD-IIV3.

We found the superior immunogenicity response for A/H1N1 strains of HD-IIV3 by integrating available evidence in the present meta-analysis; however, there was no difference in immunogenicity in terms of A/H3N2 and B strains. Furthermore, the study supported the need for larger RCTs to explore relevant clinical outcomes such as the incidence of influenza and influenza-like illness and mortality in immunocompromised patients.

### Limitations of the Study

The present meta-analysis also had inherent potential limitations. Few RCTs have examined the clinical outcomes of two different two influenza vaccine dosages in immunocompromised patients; therefore, achieving sufficient statistical power might be difficult, and a cautious approach in interpreting the results is warranted. There was substantial heterogeneity in the design, protocols, and data analyses of the included studies, some of which could have introduced bias. Moreover, there was a high degree of statistical heterogeneity observed in several immunogenicity measurements. This heterogeneity is likely due to the changes in circulating strains and variability in severity on a year-to-year basis. In general, a larger treatment effect was observed in seasons in which the A/H3N2 strain was dominant. Based on US Centers for Disease Control and Prevention (CDC) virologic surveillance, the A/H3N2 strain was dominant in the 2010–11, 2011–12, 2012–13, and 2014–15 seasons [41].

An impact on the treatment effect between A/H3N2-dominant and A/H1N1-dominant seasons was observed in several studies. In studies by Shay et al. and Gravenstein et al., there was noticeably higher vaccine efficacy in the A/H3N2 seasons than in the A/H1N1 seasons [38,39,42]. These observations suggest that differences in circulating strains may explain the variability in vaccine efficacy estimates among the studies, and future work can examine the impact of the dominant influenza strain on vaccine immunogenicity responses. Though the match of the vaccine to the circulating strain could also be an important factor, CDC virologic surveillance data suggest that, excluding the 2009–2010 and 2014–2015 seasons, the vaccine strains were generally extremely well matched to the circulating strains, indicating that mismatch was not an influential factor for vaccine immunogenicity responses. Lastly, there was variability in post-vaccination serum collection (between three and six weeks). Whether this variability contributed to differences in vaccine immunogenicity across studies is unclear.

## 5. Conclusions

Alternative higher-dose vaccination strategies appear to associate with superior immunogenicity responses for A/H1N1 strains, and the strategies were generally well tolerated in immunocompromised populations. Future studies should focus on clarifying the optimal timing, frequency, and dose, as well as assessing whether these strategies improve vaccine immunogenicity and clinical outcomes.

## Figures and Tables

**Table 1:** Basic characteristics of included randomized trials.

Author Year	Design (Country)	Duration (Strains *)	Population	Case (Mean Age)	Vaccine Doseage	Time to Vaccine	Follow up (Quality *)
Giaquinta 2015 [11] (NCT01525004)	DB, Phase I RCT, (US)	2011–2012 (A/H3N2)	Pediatric SOTR (kidney, liver, heart, lungs, intestine)	38 (12.8)	HD (60μg HA)	Post-transplant for 6 months	4 weeks (7)
McManus 2014 [15] (NCT01216332)	DB, Phase I RCT, (US)	2010–2011 (A/H3N2) 2011–2012 (A/H3N2)	Pediatric patients with Acute lymphocytic leukemia	50 (8.5)	HD (60μg HA)	Under C/T for 1 month	4 weeks (6)
Halasa 2016 [13] (NCT01215734)	DB, Phase I, RCT (US)	2010–201 1(A/H3N2) 2011–2012 (A/H3N2)	Adult Stem Cell Hematopoietic Transplant Recipients	44 (50.0)	HD (60μg HA)	Post-transplant for 6 months	4 weeks (7)
Hakim 2016 [12] (NCT01205581)	OP, Phase II, RCT (US)	2010–2011 (A/H3N2) 2011–2012 (A/H3N2)	Children with Cancer	44 (11.3)	HD (60μg HA)	Under C/T or received C/T in the past 3 months	3 weeks (5)
Jamshed 2016 [14] (NCT01666782)	DB, Phase II, RCT (US)	2012–2013 (A/H3N2) 2013–2014 (A/H1N1)	Adult with Cancer	105 (53.4)	HD (60μg HA)	First day of chemotherapy	4 weeks (6)
Cordero 2017 [10] (EudraCT 2011-003243-21)	OP, Phase III, RCT (Spanish)	2012–2013 (A/H3N2)	Adult SOTR (kidney, liver, heart, lungs)	499 (55.9)	Booster (30μg HA)	Post-transplant for 1 month	10 weeks (5)
Natori 2018 [7] (NCT03139565)	DB, Phase III, RCT (Canada)	2016–2017 (A/H3N2)	Adult SOTR (kidney, liver, heart, lung and pancreas)	161 (57)	HD (60μg HA)	Post-transplant for 3 months	4 weeks (6)
Mombelli 2018 [16] (NCT02746783)	OP, Phase II, RCT (Switzerland)	2014–2015 (A/H3N2)	Adult SOTR (kidney, liver)	79 (58.6)	DD (30μg HA)	Post-transplant for 3 months	4 weeks (5)

DB: Double blind; OP: Open label; RCT: Randomized control trial; SOTR: Solid organ transplant recipients; HA: Hemagglutinin antigen; C/T: Chemotherapy. *: Cochrane risk of bias 1.0.

**Table 2:** Meta-analyses of immunogenicity outcomes and sub-group analyses.

Outcome Assessment	H1N1			H3N2			B		
	Number Trials (Patients)	Risk Difference (95% CI)	I^2^ (%) *p*-Value	Number Trials (Patients)	Risk Ratio (95% CI)	I^2^ (%) *p*-Value	Number Trials (Patients)	Risk Ratio (95% CI)	I^2^ (%) *p*-Value
**SeroConversion**	8 (921)	0.1278 (0.0347; 0.2208)	48.7% 0.0071 *	8 (921)	0.1000 (-0.0140; 0.2141)	65.7% 0.0856	8 (921)	−0.0004 (−0.0999; 0.0991)	63.7% 0.9938
Double does (30 µg)	2 (503)	0.0483 (−0.0195; 0.1161)	0.0% 0.1625	2 (503)	0.0327 (−0.0374; 0.1028)	0.0% 0.3603	2 (503)	0.0424 (−0.0301; 0.1149)	0.0% 0.2514
High does (60 µg)	6 (418)	0.1801 (0.0651; 0.2952)	31.8% 0.0021 *	6 (418)	0.1300 (−0.0349; 0.2950)	66.9% 0.1223	6 (418)	−0.0225 (−0.1865; 0.1416)	73.6% 0.7883
Mean Age > 18	5 (804)	0.1236 (0.0310; 0.2162)	44.4% 0.0088 *	5 (804)	0.1164 (0.0088; 0.2240)	56.3% 0.0340 *	5 (804)	−0.0043 (−0.1406; 0.1319)	76.3% 0.9503
Mean Age < 18	3 (117)	0.1094 (−0.1916; 0.4105)	68.6% 0.4760	3 (117)	0.0548 (−0.3253; 0.4349)	82.0% 0.7776	3 (117)	0.0182 (−0.1292; 0.1657)	20.4% 0.8083
Chemotherapy	3 (180)	0.1386 (−0.1294; 0.4066)	72.3% 0.3107	3 (180)	0.0044 (−0.2737; 0.2825)	74.3% 0.9754	3 (180)	0.1098 (0.0080; 0.2115)	0.0% 0.0344 *
Post−transplant	5 (739)	0.0920 (0.0226; 0.1615)	11.0% 0.0093 *	5 (739)	0.1392 (0.0021; 0.2763)	67.9% 0.0466 *	5 (739)	−0.0608 (−0.1972; 0.0756)	70.2% 0.3821
**SeroProtection**	8 (921)	0.0713 (0.0209; 0.1217)	0.0% 0.0056 *	8 (921)	0.0638 (−0.0092; 0.1368)	50.2% 0.0868	8 (921)	0.0709 (−0.0230; 0.1647)	64.1% 0.1388
Double does (30 µg)	2 (503)	0.1212 (0.0404; 0.2020)	0.0% 0.0033 *	2 (503)	0.0976 (0.0268; 0.1684)	0.0% 0.0068 *	2 (503)	0.0961 (0.0309; 0.1613)	0.0% 0.0038 *
High does (60 µg)	6 (418)	0.0395 (−0.0250; 0.1040)	0.0% 0.2303	6 (418)	0.0505 (−0.0571; 0.1581)	53.9% 0.3579	6 (418)	0.0514 (−0.1139; 0.2168)	72.2% 0.5420
Mean Age > 18	5 (804)	0.0767 (0.0224; 0.1309)	0.0% 0.0056 *	5 (804)	0.0903 (−0.0025; 0.1832)	68.3% 0.0565	5 (804)	0.1094 (0.0090; 0.2099)	69.8% 0.0327 *
Mean Age < 18	3 (117)	0.0374 (−0.0987; 0.1735)	0.0% 0.5898	3 (117)	−0.0200 (−0.1580; 0.1180)	0.0% 0.7767	3 (117)	−0.0677 (−0.2800; 0.1445)	37.5% 0.5317
Chemotherapy	3 (180)	0.0365 (−0.0496; 0.1227)	0.0% 0.4061	3 (180)	−0.0101 (−0.0798; 0.0596)	0.0% 0.7759	3 (180)	0.0788 (−0.2991; 0.4568)	86.6% 0.6827
Post-transplant	5 (739)	0.0894 (0.0272; 0.1515)	0.0% 0.0048 *	5 (739)	0.1084 (0.0272; 0.1897)	36.9% 0.0089 *	5 (739)	0.0672 (0.0175; 0.1170)	0.0% 0.0080 *

SeroCoversion: Four-fold or greater rise in hemagglutination-inhibition antigen antibody titer; SeroProtection: Hemagglutination-inhibition antibody titers ≥1:40; CI: Confidence interval; I^2^: Index for assessing heterogeneity; value >50% indicates a moderate to high heterogeneity. *: The significance level in the classical model was set as < 0.5

**Table 3:** Meta-analyses of safety outcomes.

Outcome Assessment	Number of Trials (Patients)	Risk Ratio (95% CI) Fixed-Effect Estimate	Risk Ratio (95% CI) Random-Effect	*p*-Value Random-Effect	Heterogeneity I^2^ (%)
Adverse Events, all	8 (1007)	0.8879 (0.8072; 0.9766)	1.0211 (0.6770; 1.5401)	0.9208	93.7%
Mild	6 (792)	0.8094 (0.7120; 0.9201)	0.8698 (0.5722; 1.3221)	0.5137	88.7%
Moderate	6 (792)	0.9003 (0.7546; 1.0741)	0.9626 (0.5165; 1.7940)	0.9044	91.5%
Severe	6 (792)	1.0360 (0.7095; 1.5129)	1.1472 (0.4112; 3.2007)	0.7931	75.6%
Serious	5 (656)	0.8787 (0.5823; 1.3259)	0.8228 (0.5514; 1.2276)	0.3394	0.0%
Rejection	3 (787)	1.3454 (0.4205; 4.3054)	1.3795 (0.3858; 4.9330)	0.6207	0.0%

CI: Confidence interval; AE: Adverse event; I^2^: Index for assessing heterogeneity; value >50% indicates a moderate to high heterogeneity.

## Supplementary Materials

The following are available online at https://www.mdpi.com/2077-0383/8/5/590/s1, Table S1: PRISMA checklist, Table S2: Search details, Table S3: Meta-analyses result of immunogenicity outcomes (Risk ratio), Figure S1: Assessment of risk of bias, Figure S2: Forest plots of subgroup analyses with variables of Dosage, Mean Age and Status in SeroConversion Outcomes, Figure S3: Forest plots of subgroup analyses with variables of Dosage, Mean Age and Status in SeroProtection Outcomes, Figure S4: Funnel plots of major outcomes in participants with diabetes for aspirin intervention trials.
