# Supplementary material for: Alternative-Dose versus Standard-Dose Trivalent Influenza Vaccines for Immunocompromised Patients: A Meta-Analysis of Randomised Control Trials
Source: J Clin Med. 2019 Apr 29;8(5):590. doi: 10.3390/jcm8050590 (PMC6571572; doi:10.3390/jcm8050590)
Supplement: Supplementary file 1 [file jcm-08-00590-s001.pdf]

Journal of
Clinical Medicine

MDPI

Alternative-dose versus standard-dose trivalent influenza vaccines for immunocompromised patients: A meta-analysis of randomised control trials

Supplementary Materials

Table S1. PRISMA checklist
Table S2. Search details
Reference list of included full-text screening studies
Figure S1. Assessment of risk of bias.
Table S3. Meta-analyses result of immunogenicity outcomes (Risk ratio).
Figure S2. Forest plots of subgroup analyses with variables of Dosage, Mean Age and Status in SeroConversion Outcomes.
Figure S3. Forest plots of subgroup analyses with variables of Dosage, Mean Age and Status in SeroProtection Outcomes.
Figure S4. Funnel plots of major outcomes in participants with diabetes for aspirin intervention trials.

Table S1. PRISMA checklist.

Section/topic	Item No	Checklist item	Reported on page No
		**Title**	
Title	1	Identify the report as a systematic review, meta-analysis, or both	1
		**Abstract**	
Structured summary	2	Provide a structured summary including, as applicable, background, objectives, data sources, study eligibility criteria, participants, interventions, study appraisal and synthesis methods, results, limitations, conclusions and implications of key findings, systematic review registration number	2
		**Introduction**	
Rationale	3	Describe the rationale for the review in the context of what is already known	3-4
Objectives	4	Provide an explicit statement of questions being addressed with reference to participants, interventions, comparisons, outcomes, and study design (PICOS)	3-4
		**Methods**	
Protocol and registration	5	Indicate if a review protocol exists, if and where it can be accessed (such as web address), and, if available, provide registration information including registration number	4
Eligibility criteria	6	Specify study characteristics (such as PICOS, length of follow-up) and report characteristics (such as years considered, language, publication status) used as criteria for eligibility, giving rationale	4-5
Information sources	7	Describe all information sources (such as databases with dates of coverage, contact with study authors to identify additional studies) in the search and date last searched	5
Search	8	Present full electronic search strategy for at least one database, including any limits used, such that it could be repeated	4<br>Appendix 3
Study selection	9	State the process for selecting studies (that is, screening, eligibility, included in systematic review, and, if applicable, included in the meta-analysis)	5
Data collection process	10	Describe method of data extraction from reports (such as piloted forms, independently, in duplicate) and any processes for obtaining and confirming data from investigators	5
Data items	11	List and define all variables for which data were sought (such as PICOS, funding sources) and any assumptions and simplifications made	5-6
Risk of bias in individual studies	12	Describe methods used for assessing risk of bias of individual studies (including specification of whether this was done at the study or outcome level), and how this information is to be used in any data synthesis	5
Summary measures	13	State the principal summary measures (such as risk ratio, difference in means).	5-7
Synthesis of results	14	Describe the methods of handling data and combining results of studies, if done, including measures of consistency (such as I^2 statistic) for each meta-analysis	5-7
Risk of bias across studies	15	Specify any assessment of risk of bias that may affect the cumulative evidence (such as publication bias, selective reporting within studies)	5-7
Additional analyses	16	Describe methods of additional analyses (such as sensitivity or subgroup analyses, meta-regression), if done, indicating which were pre-specified	7
		**Results**	
Study selection	17	Give numbers of studies screened, assessed for eligibility, and included in the review, with reasons for exclusions at each stage, ideally with a flow diagram	7, Figure 1
Study characteristics	18	For each study, present characteristics for which data were extracted (such as study size, PICOS, follow-up period) and provide the citations	7-10, Table 1
Risk of bias within studies	19	Present data on risk of bias of each study and, if available, any outcome-level assessment (see item 12).	7-10, Appendix 5<br>Appendix 8

Results of individual studies	20	For all outcomes considered (benefits or harms), present for each study (a) simple summary data for each intervention group and (b) effect estimates and confidence intervals, ideally with a forest plot	7-10, Appendix 5
Synthesis of results	21	Present results of each meta-analysis done, including confidence intervals and measures of consistency	10-11, Figures 2-3; Table 2-2
Risk of bias across studies	22	Present results of any assessment of risk of bias across studies (see item 15)	10-11, Figures 2-3; Table 2-2
Additional analysis	23	Give results of additional analyses, if done (such as sensitivity or subgroup analyses, meta-regression) (see item 16)	10-11, Figures 2-3; Table 2-2
		**Discussion**	
Summary of evidence	24	Summarise the main findings including the strength of evidence for each main outcome; consider their relevance to key groups (such as health care providers, users, and policy makers)	11-13
Limitations	25	Discuss limitations at study and outcome level (such as risk of bias), and at review level (such as incomplete retrieval of identified research, reporting bias)	12
Conclusions	26	Provide a general interpretation of the results in the context of other evidence, and implications for future research	13
		**Funding**	
Funding	27	Describe sources of funding for the systematic review and other support (such as supply of data) and role of funders for the systematic review	None

Table S2. Search details.

Ovid MEDLINE	EMBASE (OVID)	Others
1 exp Influenza Vaccines/<br>2 Influenza, Human/ep [Epidemiology]<br>3 Influenza, Human/im [Immunology]<br>4 Influenza, Human/mo [Mortality]<br>5 Influenza, Human/pc [Prevention & Control]<br>6 Influenza, Human/tm [Transmission]<br>7 influenza vaccin$.ti,ab.<br>8 (influenza or flu).ti,ab.<br>9 (vaccin$ or immuni$ or inocul$ or efficacy or effectiveness).ti,ab.<br>10 and/8-9<br>11 or/1-7,10<br>12 RANDOMIZED CONTROLLED TRIAL.pt.<br>13 CONTROLLED CLINICAL TRIAL.pt.<br>14 RANDOMIZED CONTROLLED TRIALS.sh.<br>15 RANDOM ALLOCATION.sh.<br>16 DOUBLE BLIND METHOD.sh.<br>17 SINGLE-BLIND METHOD.sh.<br>18 or/12-17<br>19 Animals/<br>20 Humans/<br>21 19 not 20<br>22 18 not 21<br>23 CLINICAL TRIAL.pt.<br>24 exp Clinical Trials/<br>25 (clin$ adj25 trial$).ti,ab.<br>26 ((singl$ or doubl$ or trebl$ or tripl$) adj25 (blind$ or mask$)).ti,ab.<br>27 PLACEBOS.sh.<br>28 placebo$.ti,ab.<br>29 random$.ti,ab.<br>30 or/23-29<br>31 30 not 21<br>32 exp Research Design/<br>33 exp Comparative Study/<br>34 exp Evaluation Studies/<br>35 exp Follow-Up Studies/<br>36 exp Prospective Studies/<br>37 prospectiv$.ti,ab.<br>38 volunteer$.ti,ab.<br>39 exp Case-Control Studies/<br>40 (cases and controls).ti,ab.<br>41 case control stud$.ti,ab.<br>42 exp Cohort Studies/<br>43 cohort stud$.ti,ab.<br>44 observational.ti,ab.<br>45 or/32-44<br>46 45 not 21<br>47 or/22,31,46<br>48 11 and 47	1. exp Influenza Vaccines/<br>2. Influenza Vaccines.ab,ti.<br>3. 1 or 2<br>4. exp transplant/<br>5. transplant.ab,ti.<br>6. 4 or 5<br>7. exp chemotherapy/<br>8. chemotherapy.ab,ti.<br>9. 7 or 8<br>10. 6 or 9<br>11. 3 and 10<br>12. cross-over procedure/ or double-blind procedure/ or randomized controlled trial/ or single-blind procedure/<br>13. (allocat* or assign* or cross over* or crossover* or (double ADJ blind*) or factorial or placebo* or random* or (single ADJ blind*) or volunteer*).ti,ab.<br>14. 13 or 14<br>15. limit 15 to animals<br>16. limit 15 to (animals and humans)<br>17. 16 not 17<br>18. 15 not 18<br>19. 12 and 19	Web of Science<br>• SET[1]: (influenza vaccine); DocType=All document types; Language=All languages;<br>• SET[2]: Refined by: TOPIC (high dose OR high-dose OR HD)<br>• SET[3]: Refined by: TOPIC (transplant* OR cancer* OR chemotherapy*)<br>Pubmed<br>• SET[1]: (influenza vaccin*)<br>• SET[2]: ("high-dose trivalent" or "high dose trivalent" or "high dose influenza" or "Fluzone high dose" or "Fluzone high-dose" or "Fluzone HD" or "high-dose TIV" or "high dose TIV" or "highdose IIV3" or "high dose IIV3" or "IIV3-HD" or “high dose” or “high-dose” or “HD”)<br>((ageda OR (aged, 80 and over) OR senior* OR geriatric* OR old* adult OR 65))<br>• SET[3]: (transplant* OR cancer* OR chemotherapy*)<br>• SET[4]: 1 and 2 and 3

Supplementary Materials: Reference list of full-text screening studies. Included studies (No.1 to No.8)

1. GiaQuinta S; Randomized, double-blind comparison of standard-dose vs. high-dose trivalent inactivated influenza vaccine in pediatric solid organ transplant patients; DOI: 10.1111/petr.12419.
2. Meghann McManus; Safety and Immunogenicity of High Dose Trivalent Inactivated Influenza Vaccine in Pediatric Patients with Acute Lymphoblastic Leukemia; doi:10.1002/pbc.24863
3. Hana Hakim;Immunogenicity and Safety of High-Dose Trivalent Inactivated Influenza Vaccine Compared to Standard-Dose Vaccine in Children and Young Adults with Cancer or HIV Infection; doi:10.1016/j.vaccine.2016.04.053.
4. Natasha B. Halasa; Randomized Double-Blind Study of the Safety and Immunogenicity of Standard-Dose Trivalent Inactivated Influenza Vaccine versus High-Dose Trivalent Inactivated Influenza Vaccine in Adult Hematopoietic Stem Cell Transplantation Patients; http://dx.doi.org/10.1016/j.bbmt.2015.12.003
5. Saad Jamsheda; Improved immunogenicity of high-dose influenza vaccine comparedto standard-dose influenza vaccine in adult oncology patientsyounger than 65 years receiving chemotherapy: A pilot randomizedclinical trial; http://dx.doi.org/10.1016/j.vaccine.2015.12.037
6. Yoichiro Natori; A Double Blind Randomized Trial of High Dose vs. Standard Dose Influenza Vaccine in Adult Solid Organ Transplant Recipients; https://academic.oup.com/cid/advance-article-abstract/doi/10.1093/cid/cix1082/4743251
7. Elisa Cordero; Two Doses of Inactivated Influenza Vaccine Improve Immune Response in Solid Organ Transplant Recipients: Results of TRANSGRIPE 1–2, a Randomized Controlled Clinical Trial; DOI: 10.1093/cid/ciw855
8. Matteo Mombelli; Immunogenicity and safety of double versus standard dose of the seasonal influenza vaccine in solid-organ transplant recipients: A randomized controlled trial; https://doi.org/10.1016/j.vaccine.2018.08.057
9. A. Baluch; Randomized Controlled Trial of High-Dose Intradermal Versus Standard-Dose Intramuscular Influenza Vaccine in Organ Transplant Recipients; doi: 10.1111/ajt.12149
10. Iva Hojsak; Antibody Response to Influenza Vaccine in Pediatric Liver Transplant Recipients; DOI: 10.1097/INF.0b013e31820b7c22
11. Nicole Le Correa; Effect of two injections of non-adjuvanted influenza A H1N1pdm2009 vaccine in renal transplant recipients: INSERM C09-32 TRANSFLUVAC trial; http://dx.doi.org/10.1016/j.vaccine.2012.10.047
12. David A. Nace; Randomized, Controlled Trial of High-Dose Influenza Vaccine Among Frail Residents of Long-Term Care Facilities; DOI: 10.1093/infdis/jiu622
13. April Sykes; The Effectiveness of Trivalent Inactivated Influenza Vaccine in Children with Acute Leukemia; doi:10.1016/j.jpeds.2017.08.071
14. Yukinari Sanada; A prospective study on the efficacy of two-dose influenza vaccinations in cancer patients receiving chemotherapy; doi: 10.1093/jjco/hyw020
15. Brydak LB, Guzy J, Starzyk J, Machala M, Gozdz SS. Humoral immune response after vaccination against influenza in patients with breast cancer. *Support Care Cancer*. 2001;9:65–8.
16. Lo W, Whimbey E, Elting L, Couch R, Cabanillas F, Bodey G. Antibody response to a two-dose influenza vaccine regimen in adult lymphoma patients on chemotherapy. *Eur J Clin Microbiol Infect Dis*. 1993;12:778–82.
17. Ramanathan RK, Potter DM, Belani CP, et al. Randomized trial of influenza vaccine with granulocyte-macrophage colony-stimulating factor or placebo in cancer patients. *J Clin Oncol*. 2002;20:4313–8.
18. Brydak LB, Machala M, Centkowski P, Warzocha K, Bilinski P. Humoral response to hemagglutinin components of influenza vaccine in patients with non-Hodgkin malignant lymphoma. *Vaccine*. 2006;24:6620–3.
19. Gribabis DA, Panayiotidis P, Boussiotis VA, Hannoun C, Pangalis GA. Influenza virus vaccine in B-cell chronic lymphocytic leukaemia patients. *Acta Haematol*. 1994;91:115–8.
20. Hodges GR, Davis JW, Lewis HD Jr, et al. Response to influenza A vaccine among high-risk patients. *South Med J*. 1979;72:29–32.

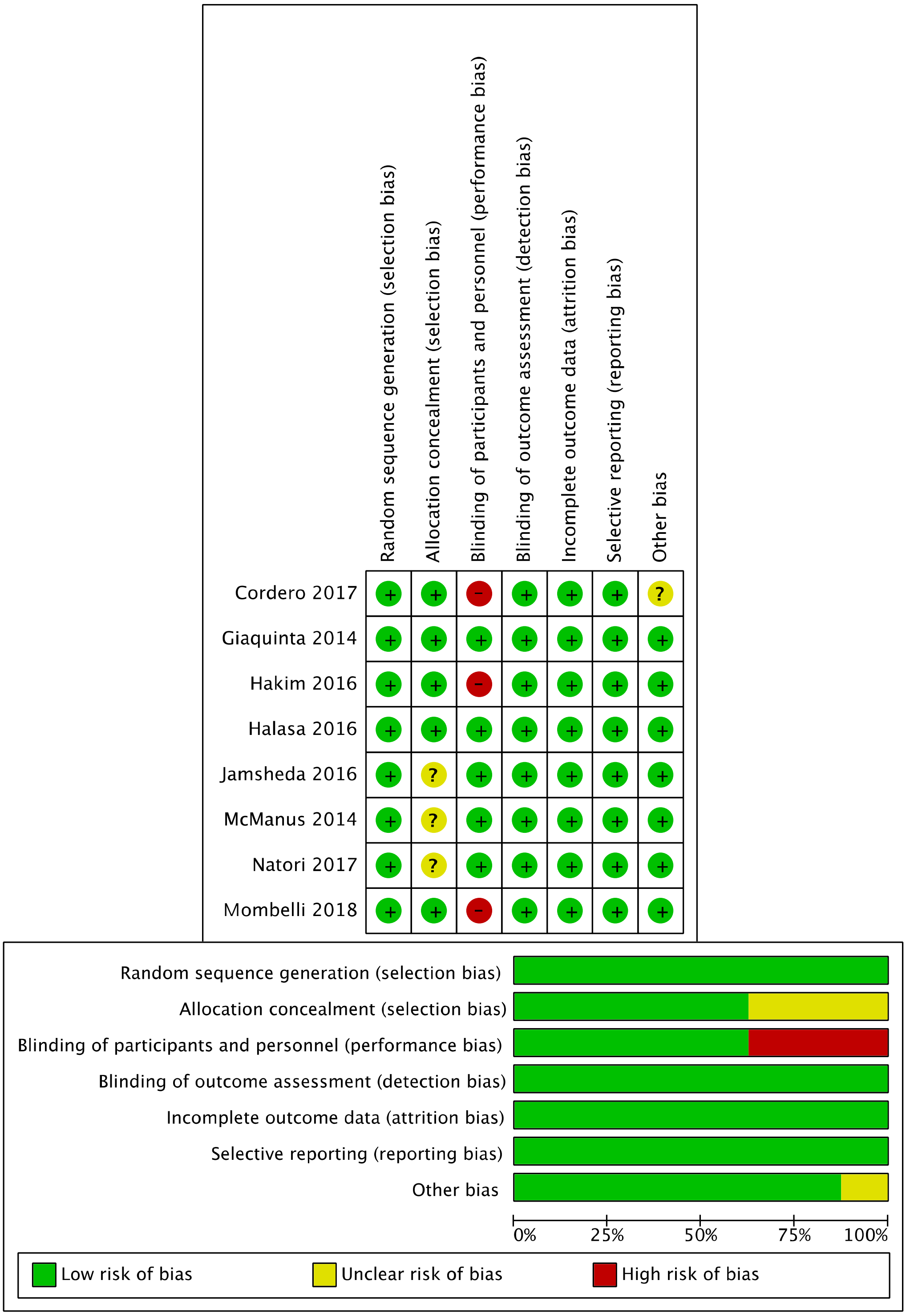

Figure S1. Assessment of risk of bias.

Table S3. Meta-analyses result of immunogenicity outcomes (Risk ratio).

Outcome assessment	No. of trials (patients)	Risk Ratio (95% CI) Fixed-Effect estimate	Risk Ratio (95% CI) Random-Effect	P-valueRandom-Effect	Heterogeneity I^2 (%)
**SeroConversion**					
H1N1	8 (921)	1.48 [1.21; 1.81]	1.45 [1.15; 1.83]	0.29	18%
H3N2	8 (921)	1.31 [1.11; 1.55]	1.23 [0.94; 1.61]	0.03	55%
B	8 (921)	0.99 [0.84; 1.15]	0.97 [0.73; 1.30]	0.02	59%
**SeroProtection**					
H1N1	8 (921)	1.12 [1.03; 1.22]	1.09 [1.01; 1.17]	0.38	7%
H3N2	8 (921)	1.13 [1.04; 1.23]	1.08 [0.96; 1.20]	0.01	62%
B	8 (921)	1.13 [1.06; 1.21]	1.10 [0.97; 1.24]	< 0.01	62%

Risk Ratio > 1 indicates immunogenicity outcome favors alternative higher dose. **SeroCoversion:** 4-fold or greater rise in hemagglutination-inhibition antigen antibody titer**; SeroProtection:** hemagglutination-inhibition antibody titers ≥1:40**; CI:** confidence interval**; I^2:** index for assessing heterogeneity; value >50% indicates a moderate to high heterogeneity.

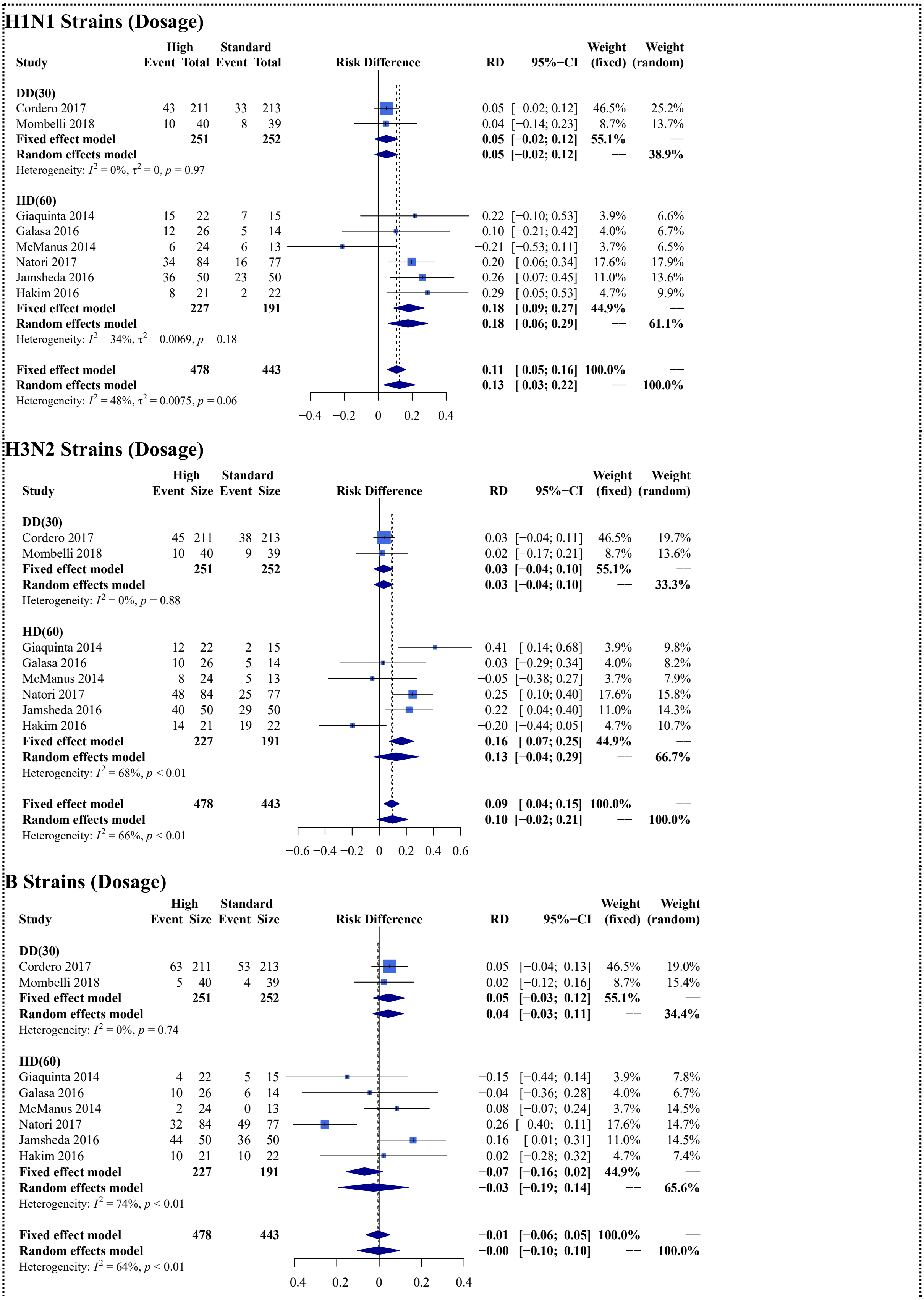
H1N1 Strains (Dosage)
Study
High Event Total
Standard Event Total
Risk Difference
RD
95%−CI
Weight (fixed)
Weight (random)
DD(30)
Cordero 2017 43 211 33 213 0.05 [−0.02; 0.12] 46.5% 25.2%
Mombelli 2018 10 40 8 39 0.04 [−0.14; 0.23] 8.7% 13.7%
Fixed effect model 251 252 0.05 [−0.02; 0.12] 55.1% —
Random effects model 0.05 [−0.02; 0.12] — 38.9%
Heterogeneity: $I^2 = 0\%$, $\tau^2 = 0$, $p = 0.97$
HD(60)
Giaquinta 2014 15 22 7 15 0.22 [−0.10; 0.53] 3.9% 6.6%
Galasa 2016 12 26 5 14 0.10 [−0.21; 0.42] 4.0% 6.7%
McManus 2014 6 24 6 13 −0.21 [−0.53; 0.11] 3.7% 6.5%
Natori 2017 34 84 16 77 0.20 [0.06; 0.34] 17.6% 17.9%
Jamsheda 2016 36 50 23 50 0.26 [0.07; 0.45] 11.0% 13.6%
Hakim 2016 8 21 2 22 0.29 [0.05; 0.53] 4.7% 9.9%
Fixed effect model 227 191 0.18 [0.09; 0.27] 44.9% —
Random effects model 0.18 [0.06; 0.29] — 61.1%
Heterogeneity: $I^2 = 34\%$, $\tau^2 = 0.0069$, $p = 0.18$
Fixed effect model 478 443 0.11 [0.05; 0.16] 100.0% —
Random effects model 0.13 [0.03; 0.22] — 100.0%
Heterogeneity: $I^2 = 48\%$, $\tau^2 = 0.0075$, $p = 0.06$
−0.4 −0.2 0 0.2 0.4
H3N2 Strains (Dosage)
Study
High Event Size
Standard Event Size
Risk Difference
RD
95%−CI
Weight (fixed)
Weight (random)
DD(30)
Cordero 2017 45 211 38 213 0.03 [−0.04; 0.11] 46.5% 19.7%
Mombelli 2018 10 40 9 39 0.02 [−0.17; 0.21] 8.7% 13.6%
Fixed effect model 251 252 0.03 [−0.04; 0.10] 55.1% —
Random effects model 0.03 [−0.04; 0.10] — 33.3%
Heterogeneity: $I^2 = 0\%$, $p = 0.88$
HD(60)
Giaquinta 2014 12 22 2 15 0.41 [0.14; 0.68] 3.9% 9.8%
Galasa 2016 10 26 5 14 0.03 [−0.29; 0.34] 4.0% 8.2%
McManus 2014 8 24 5 13 −0.05 [−0.38; 0.27] 3.7% 7.9%
Natori 2017 48 84 25 77 0.25 [0.10; 0.40] 17.6% 15.8%
Jamsheda 2016 40 50 29 50 0.22 [0.04; 0.40] 11.0% 14.3%
Hakim 2016 14 21 19 22 −0.20 [−0.44; 0.05] 4.7% 10.7%
Fixed effect model 227 191 0.16 [0.07; 0.25] 44.9% —
Random effects model 0.13 [−0.04; 0.29] — 66.7%
Heterogeneity: $I^2 = 68\%$, $p < 0.01$
Fixed effect model 478 443 0.09 [0.04; 0.15] 100.0% —
Random effects model 0.10 [−0.02; 0.21] — 100.0%
Heterogeneity: $I^2 = 66\%$, $p < 0.01$
−0.6 −0.4 −0.2 0 0.2 0.4 0.6
B Strains (Dosage)
Study
High Event Size
Standard Event Size
Risk Difference
RD
95%−CI
Weight (fixed)
Weight (random)
DD(30)
Cordero 2017 63 211 53 213 0.05 [−0.04; 0.13] 46.5% 19.0%
Mombelli 2018 5 40 4 39 0.02 [−0.12; 0.16] 8.7% 15.4%
Fixed effect model 251 252 0.05 [−0.03; 0.12] 55.1% —
Random effects model 0.04 [−0.03; 0.11] — 34.4%
Heterogeneity: $I^2 = 0\%$, $p = 0.74$
HD(60)
Giaquinta 2014 4 22 5 15 −0.15 [−0.44; 0.14] 3.9% 7.8%
Galasa 2016 10 26 6 14 −0.04 [−0.36; 0.28] 4.0% 6.7%
McManus 2014 2 24 0 13 0.08 [−0.07; 0.24] 3.7% 14.5%
Natori 2017 32 84 49 77 −0.26 [−0.40; −0.11] 17.6% 14.7%
Jamsheda 2016 44 50 36 50 0.16 [0.01; 0.31] 11.0% 14.5%
Hakim 2016 10 21 10 22 0.02 [−0.28; 0.32] 4.7% 7.4%
Fixed effect model 227 191 −0.07 [−0.16; 0.02] 44.9% —
Random effects model −0.03 [−0.19; 0.14] — 65.6%
Heterogeneity: $I^2 = 74\%$, $p < 0.01$
Fixed effect model 478 443 −0.01 [−0.06; 0.05] 100.0% —
Random effects model −0.00 [−0.10; 0.10] — 100.0%
Heterogeneity: $I^2 = 64\%$, $p < 0.01$
−0.4 −0.2 0 0.2 0.4

H1N1 Strains (Mean Age)

Study	High Event	High Total	Standard Event	Standard Total	RD	95%−CI	Weight (fixed)	Weight (random)
**Mean Age<18 years**								
Giaquinta 2014	15	22	7	15	0.22	[−0.10; 0.53]	3.9%	6.6%
McManus 2014	6	24	6	13	−0.21	[−0.53; 0.11]	3.7%	6.5%
Hakim 2016	8	21	2	22	0.29	[0.05; 0.53]	4.7%	9.9%
**Fixed effect model**		**67**		**50**	**0.12**	**[−0.05; 0.28]**	**12.3%**	--
**Random effects model**					**0.11**	**[−0.19; 0.41]**	--	**23.0%**
Heterogeneity: $I^2 = 69\%$, $\tau^2 = 0.0484$, $p = 0.04$								
**Mean Age>18 years**								
Cordero 2017	43	211	33	213	0.05	[−0.02; 0.12]	46.5%	25.2%
Galasa 2016	12	26	5	14	0.10	[−0.21; 0.42]	4.0%	6.7%
Mombelli 2018	10	40	8	39	0.04	[−0.14; 0.23]	8.7%	13.7%
Natori 2017	34	84	16	77	0.20	[0.06; 0.34]	17.6%	17.9%
Jamsheda 2016	36	50	23	50	0.26	[0.07; 0.45]	11.0%	13.6%
**Fixed effect model**		**411**		**393**	**0.11**	**[0.05; 0.16]**	**87.7%**	--
**Random effects model**					**0.12**	**[0.03; 0.21]**	--	**77.0%**
Heterogeneity: $I^2 = 44\%$, $\tau^2 = 0.0045$, $p = 0.13$								
**Fixed effect model**		**478**		**443**	**0.11**	**[0.05; 0.16]**	**100.0%**	--
**Random effects model**					**0.13**	**[0.03; 0.22]**	--	**100.0%**
Heterogeneity: $I^2 = 48\%$, $\tau^2 = 0.0075$, $p = 0.06$								

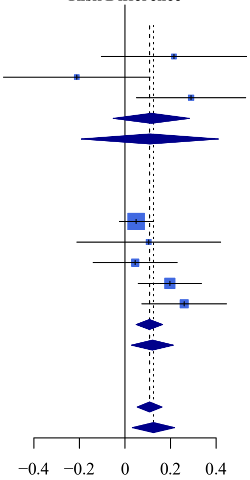

H3N2 Strains (Mean Age)

Study	High Event	High Size	Standard Event	Standard Size	RD	95%−CI	Weight (fixed)	Weight (random)
**Mean Age<18 years**								
Giaquinta 2014	12	22	2	15	0.41	[0.14; 0.68]	3.9%	9.8%
McManus 2014	8	24	5	13	−0.05	[−0.38; 0.27]	3.7%	7.9%
Hakim 2016	14	21	19	22	−0.20	[−0.44; 0.05]	4.7%	10.7%
**Fixed effect model**		**67**		**50**	**0.04**	**[−0.12; 0.20]**	**12.3%**	--
**Random effects model**					**0.05**	**[−0.33; 0.43]**	--	**28.4%**
Heterogeneity: $I^2 = 82\%$, $p < 0.01$								
**Mean Age>18 years**								
Cordero 2017	45	211	38	213	0.03	[−0.04; 0.11]	46.5%	19.7%
Galasa 2016	10	26	5	14	0.03	[−0.29; 0.34]	4.0%	8.2%
Mombelli 2018	10	40	9	39	0.02	[−0.17; 0.21]	8.7%	13.6%
Natori 2017	48	84	25	77	0.25	[0.10; 0.40]	17.6%	15.8%
Jamsheda 2016	40	50	29	50	0.22	[0.04; 0.40]	11.0%	14.3%
**Fixed effect model**		**411**		**393**	**0.10**	**[0.04; 0.16]**	**87.7%**	--
**Random effects model**					**0.11**	**[0.01; 0.22]**	--	**71.6%**
Heterogeneity: $I^2 = 57\%$, $p = 0.06$								
**Fixed effect model**		**478**		**443**	**0.09**	**[0.04; 0.15]**	**100.0%**	--
**Random effects model**					**0.10**	**[−0.02; 0.21]**	--	**100.0%**
Heterogeneity: $I^2 = 66\%$, $p < 0.01$								

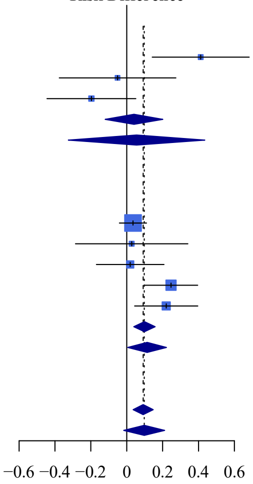

B Strains (Mean Age)

Study	High Event	High Size	Standard Event	Standard Size	RD	95%−CI	Weight (fixed)	Weight (random)
**Mean Age<18 years**								
Giaquinta 2014	4	22	5	15	−0.15	[−0.44; 0.14]	3.9%	7.8%
McManus 2014	2	24	0	13	0.08	[−0.07; 0.24]	3.7%	14.5%
Hakim 2016	10	21	10	22	0.02	[−0.28; 0.32]	4.7%	7.4%
**Fixed effect model**		**67**		**50**	**−0.01**	**[−0.17; 0.14]**	**12.3%**	--
**Random effects model**					**0.02**	**[−0.13; 0.17]**	--	**29.7%**
Heterogeneity: $I^2 = 20\%$, $p = 0.28$								
**Mean Age>18 years**								
Cordero 2017	63	211	53	213	0.05	[−0.04; 0.13]	46.5%	19.0%
Galasa 2016	10	26	6	14	−0.04	[−0.36; 0.28]	4.0%	6.7%
Mombelli 2018	5	40	4	39	0.02	[−0.12; 0.16]	8.7%	15.4%
Natori 2017	32	84	49	77	−0.26	[−0.40; −0.11]	17.6%	14.7%
Jamsheda 2016	44	50	36	50	0.16	[0.01; 0.31]	11.0%	14.5%
**Fixed effect model**		**411**		**393**	**−0.00**	**[−0.07; 0.06]**	**87.7%**	--
**Random effects model**					**−0.01**	**[−0.14; 0.13]**	--	**70.3%**
Heterogeneity: $I^2 = 76\%$, $p < 0.01$								
**Fixed effect model**		**478**		**443**	**−0.01**	**[−0.06; 0.05]**	**100.0%**	--
**Random effects model**					**−0.00**	**[−0.10; 0.10]**	--	**100.0%**
Heterogeneity: $I^2 = 64\%$, $p < 0.01$								

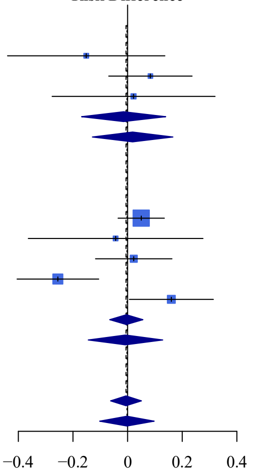

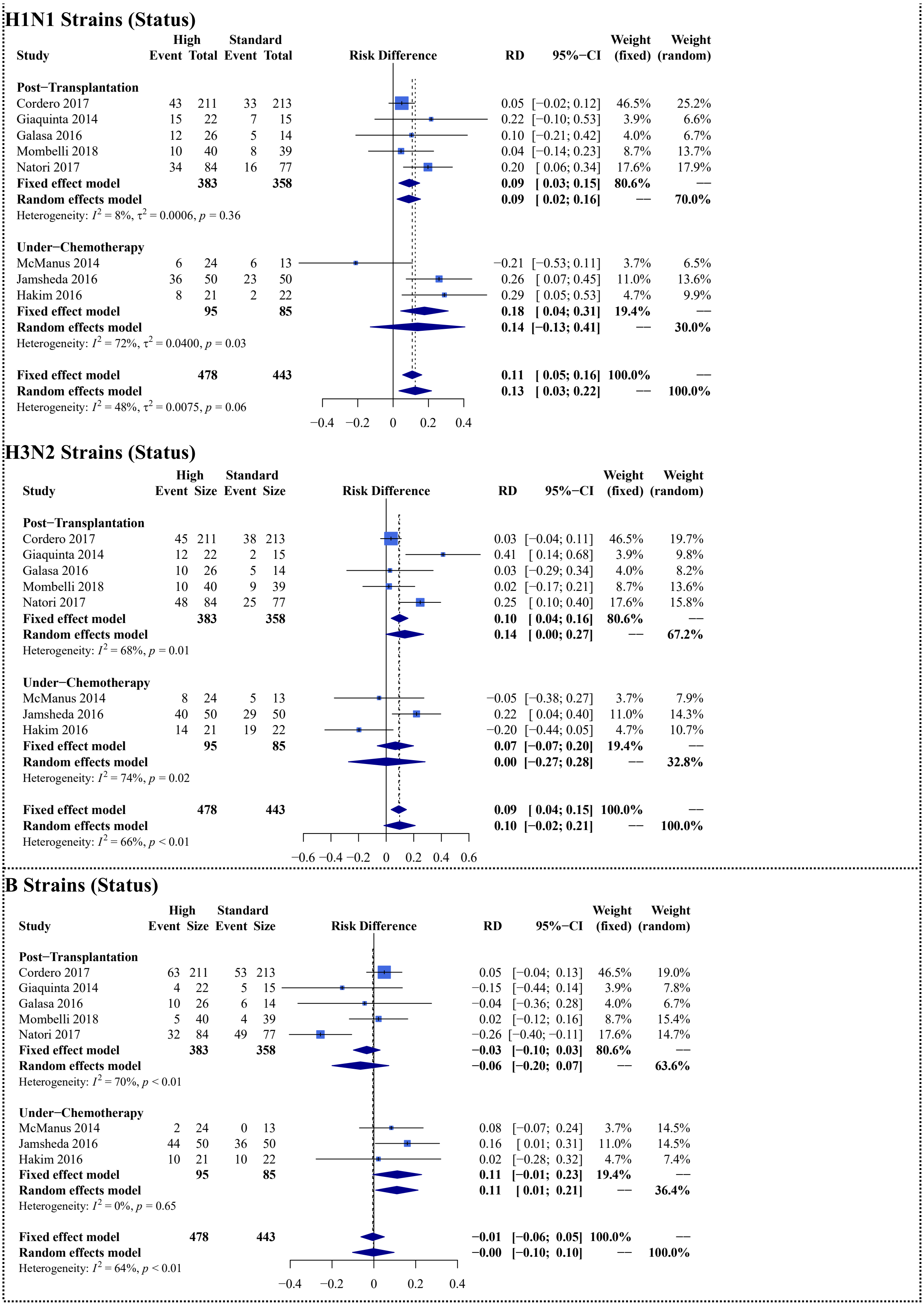

Figure S2. Forest plots of subgroup analyses with variables of Dosage, Mean Age and Status in SeroConversion Outcomes.

H1N1 Strains (Dosage)

Study	High Event	High Size	Standard Event	Standard Size	RD	95%−CI	Weight (fixed)	Weight (random)
**DD(30)**								
Cordero 2017	114	211	92	213	0.11	[0.01; 0.20]	46.5%	28.4%
Mombelli 2018	37	40	30	39	0.16	[0.00; 0.31]	8.7%	10.5%
**Fixed effect model**		**251**		**252**	**0.12**	**[0.03; 0.20]**	**55.1%**	—
**Random effects model**					**0.12**	**[0.04; 0.20]**	—	**38.9%**
Heterogeneity: $I^2 = 0\%$, $p = 0.60$								
**HD(60)**								
Giaquinta 2014	21	22	12	15	0.15	[−0.07; 0.37]	3.9%	5.2%
Galasa 2016	18	26	8	14	0.12	[−0.19; 0.43]	4.0%	2.6%
McManus 2014	20	24	11	13	−0.01	[−0.26; 0.23]	3.7%	4.2%
Natori 2017	70	84	64	77	0.00	[−0.11; 0.12]	17.6%	19.0%
Jamsheda 2016	48	50	45	50	0.06	[−0.04; 0.16]	11.0%	25.8%
Hakim 2016	16	21	18	22	−0.06	[−0.30; 0.19]	4.7%	4.3%
**Fixed effect model**		**227**		**191**	**0.03**	**[−0.04; 0.10]**	**44.9%**	—
**Random effects model**					**0.04**	**[−0.03; 0.10]**	—	**61.1%**
Heterogeneity: $I^2 = 0\%$, $p = 0.75$								
**Fixed effect model**		**478**		**443**	**0.08**	**[0.02; 0.13]**	**100.0%**	—
**Random effects model**					**0.07**	**[0.02; 0.12]**	—	**100.0%**
Heterogeneity: $I^2 = 0\%$, $p = 0.61$								

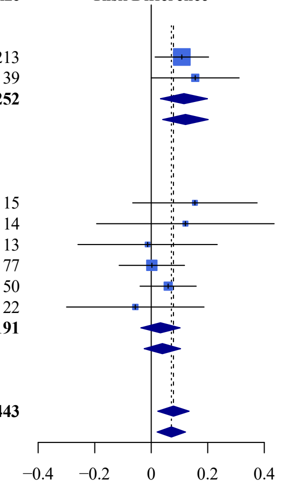

H3N2 Strains (Dosage)

Study	High Event	High Size	Standard Event	Standard Size	RD	95%−CI	Weight (fixed)	Weight (random)
**DD(30)**								
Cordero 2017	120	211	97	213	0.11	[0.02; 0.21]	46.5%	19.5%
Mombelli 2018	39	40	35	39	0.08	[−0.03; 0.18]	8.7%	17.9%
**Fixed effect model**		**251**		**252**	**0.11**	**[0.03; 0.19]**	**55.1%**	—
**Random effects model**					**0.10**	**[0.03; 0.17]**	—	**37.4%**
Heterogeneity: $I^2 = 0\%$, $p = 0.57$								
**HD(60)**								
Giaquinta 2014	19	22	12	15	0.06	[−0.18; 0.31]	3.9%	6.7%
Galasa 2016	21	26	5	14	0.45	[0.16; 0.74]	4.0%	5.1%
McManus 2014	13	24	8	13	−0.07	[−0.40; 0.26]	3.7%	4.2%
Natori 2017	67	84	57	77	0.06	[−0.07; 0.19]	17.6%	15.1%
Jamsheda 2016	48	50	48	50	0.00	[−0.08; 0.08]	11.0%	21.9%
Hakim 2016	18	21	20	22	−0.05	[−0.24; 0.14]	4.7%	9.7%
**Fixed effect model**		**227**		**191**	**0.06**	**[−0.02; 0.13]**	**44.9%**	—
**Random effects model**					**0.05**	**[−0.06; 0.16]**	—	**62.6%**
Heterogeneity: $I^2 = 54\%$, $p = 0.05$								
**Fixed effect model**		**478**		**443**	**0.08**	**[0.03; 0.14]**	**100.0%**	—
**Random effects model**					**0.06**	**[−0.01; 0.14]**	—	**100.0%**
Heterogeneity: $I^2 = 50\%$, $p = 0.05$								

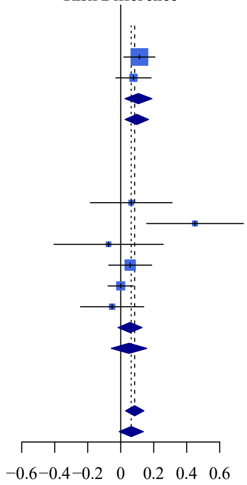

B Strains (Dosage)

Study	High Event	High Size	Standard Event	Standard Size	RD	95%−CI	Weight (fixed)	Weight (random)
**DD(30)**								
Cordero 2017	176	211	153	213	0.12	[0.04; 0.19]	46.5%	20.7%
Mombelli 2018	38	40	35	39	0.05	[−0.06; 0.17]	8.7%	17.6%
**Fixed effect model**		**251**		**252**	**0.11**	**[0.04; 0.17]**	**55.1%**	—
**Random effects model**					**0.10**	**[0.03; 0.16]**	—	**38.3%**
Heterogeneity: $I^2 = 0\%$, $p = 0.35$								
**HD(60)**								
Giaquinta 2014	10	22	7	15	−0.01	[−0.34; 0.32]	3.9%	6.1%
Galasa 2016	11	26	6	14	−0.01	[−0.33; 0.32]	4.0%	6.3%
McManus 2014	19	24	9	13	0.10	[−0.20; 0.40]	3.7%	7.0%
Natori 2017	79	84	70	77	0.03	[−0.05; 0.11]	17.6%	20.4%
Jamsheda 2016	40	50	22	50	0.36	[0.18; 0.54]	11.0%	13.0%
Hakim 2016	13	21	19	22	−0.24	[−0.50; 0.01]	4.7%	8.8%
**Fixed effect model**		**227**		**191**	**0.08**	**[0.00; 0.16]**	**44.9%**	—
**Random effects model**					**0.05**	**[−0.11; 0.22]**	—	**61.7%**
Heterogeneity: $I^2 = 72\%$, $p < 0.01$								
**Fixed effect model**		**478**		**443**	**0.09**	**[0.04; 0.15]**	**100.0%**	—
**Random effects model**					**0.07**	**[−0.02; 0.16]**	—	**100.0%**
Heterogeneity: $I^2 = 64\%$, $p < 0.01$								

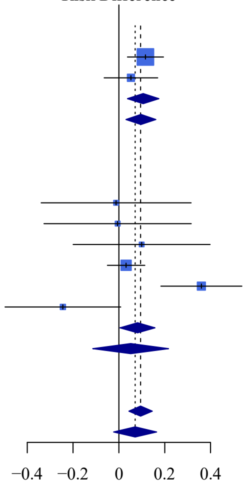

H1N1 Strains (Mean Age)

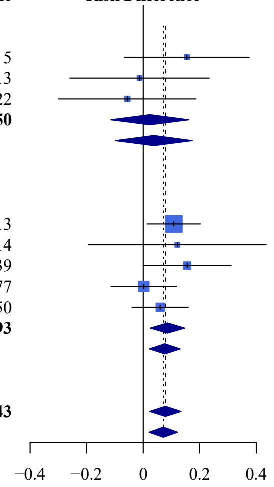

Study	High Event	High Size	Standard Event	Standard Size	RD	95%−CI	Weight (fixed)	Weight (random)
**Mean Age<18 years**								
Giaquinta 2014	21	22	12	15	0.15	[−0.07; 0.37]	3.9%	5.2%
McManus 2014	20	24	11	13	−0.01	[−0.26; 0.23]	3.7%	4.2%
Hakim 2016	16	21	18	22	−0.06	[−0.30; 0.19]	4.7%	4.3%
**Fixed effect model**		**67**		**50**	**0.02**	**[−0.11; 0.16]**	**12.3%**	—
**Random effects model**					**0.04**	**[−0.10; 0.17]**	—	**13.7%**
Heterogeneity: $I^2 = 0\%$, $p = 0.40$								
**Mean Age>18 years**								
Cordero 2017	114	211	92	213	0.11	[0.01; 0.20]	46.5%	28.4%
Galasa 2016	18	26	8	14	0.12	[−0.19; 0.43]	4.0%	2.6%
Mombelli 2018	37	40	30	39	0.16	[0.00; 0.31]	8.7%	10.5%
Natori 2017	70	84	64	77	0.00	[−0.11; 0.12]	17.6%	19.0%
Jamsheda 2016	48	50	45	50	0.06	[−0.04; 0.16]	11.0%	25.8%
**Fixed effect model**		**411**		**393**	**0.09**	**[0.03; 0.15]**	**87.7%**	—
**Random effects model**					**0.08**	**[0.02; 0.13]**	—	**86.3%**
Heterogeneity: $I^2 = 0\%$, $p = 0.50$								
**Fixed effect model**		**478**		**443**	**0.08**	**[0.02; 0.13]**	**100.0%**	—
**Random effects model**					**0.07**	**[0.02; 0.12]**	—	**100.0%**
Heterogeneity: $I^2 = 0\%$, $p = 0.61$								

H3N2 Strains (Mean Age)

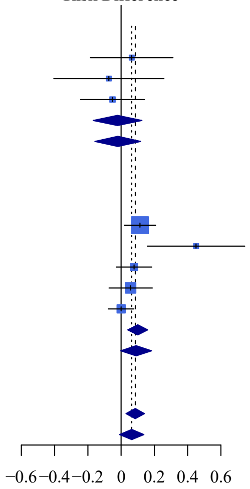

Study	High Event	High Size	Standard Event	Standard Size	RD	95%−CI	Weight (fixed)	Weight (random)
**Mean Age<18 years**								
Giaquinta 2014	19	22	12	15	0.06	[−0.18; 0.31]	3.9%	6.7%
McManus 2014	13	24	8	13	−0.07	[−0.40; 0.26]	3.7%	4.2%
Hakim 2016	18	21	20	22	−0.05	[−0.24; 0.14]	4.7%	9.7%
**Fixed effect model**		**67**		**50**	**−0.02**	**[−0.17; 0.12]**	**12.3%**	—
**Random effects model**					**−0.02**	**[−0.16; 0.12]**	—	**20.5%**
Heterogeneity: $I^2 = 0\%$, $p = 0.72$								
**Mean Age>18 years**								
Cordero 2017	120	211	97	213	0.11	[0.02; 0.21]	46.5%	19.5%
Galasa 2016	21	26	5	14	0.45	[0.16; 0.74]	4.0%	5.1%
Mombelli 2018	39	40	35	39	0.08	[−0.03; 0.18]	8.7%	17.9%
Natori 2017	67	84	57	77	0.06	[−0.07; 0.19]	17.6%	15.1%
Jamsheda 2016	48	50	48	50	0.00	[−0.08; 0.08]	11.0%	21.9%
**Fixed effect model**		**411**		**393**	**0.10**	**[0.04; 0.16]**	**87.7%**	—
**Random effects model**					**0.09**	**[0.00; 0.18]**	—	**79.5%**
Heterogeneity: $I^2 = 68\%$, $p = 0.01$								
**Fixed effect model**		**478**		**443**	**0.08**	**[0.03; 0.14]**	**100.0%**	—
**Random effects model**					**0.06**	**[−0.01; 0.14]**	—	**100.0%**
Heterogeneity: $I^2 = 50\%$, $p = 0.05$								

B Strains (Mean Age)

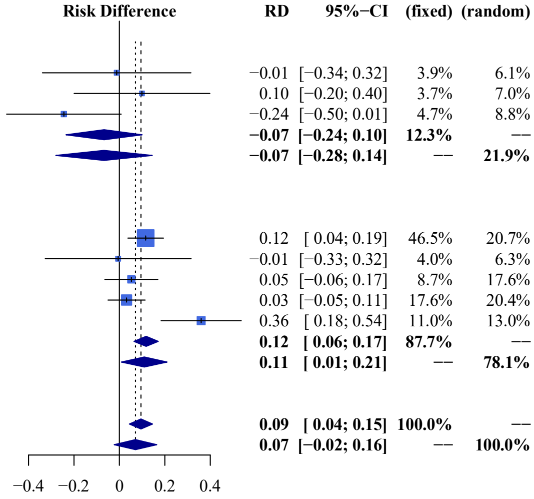

Study	High Event	High Size	Standard Event	Standard Size	RD	95%−CI	Weight (fixed)	Weight (random)
**Mean Age<18 years**								
Giaquinta 2014	10	22	7	15	−0.01	[−0.34; 0.32]	3.9%	6.1%
McManus 2014	19	24	9	13	0.10	[−0.20; 0.40]	3.7%	7.0%
Hakim 2016	13	21	19	22	−0.24	[−0.50; 0.01]	4.7%	8.8%
**Fixed effect model**		**67**		**50**	**−0.07**	**[−0.24; 0.10]**	**12.3%**	—
**Random effects model**					**−0.07**	**[−0.28; 0.14]**	—	**21.9%**
Heterogeneity: $I^2 = 37\%$, $p = 0.20$								
**Mean Age>18 years**								
Cordero 2017	176	211	153	213	0.12	[0.04; 0.19]	46.5%	20.7%
Galasa 2016	11	26	6	14	−0.01	[−0.33; 0.32]	4.0%	6.3%
Mombelli 2018	38	40	35	39	0.05	[−0.06; 0.17]	8.7%	17.6%
Natori 2017	79	84	70	77	0.03	[−0.05; 0.11]	17.6%	20.4%
Jamsheda 2016	40	50	22	50	0.36	[0.18; 0.54]	11.0%	13.0%
**Fixed effect model**		**411**		**393**	**0.12**	**[0.06; 0.17]**	**87.7%**	—
**Random effects model**					**0.11**	**[0.01; 0.21]**	—	**78.1%**
Heterogeneity: $I^2 = 70\%$, $p = 0.01$								
**Fixed effect model**		**478**		**443**	**0.09**	**[0.04; 0.15]**	**100.0%**	—
**Random effects model**					**0.07**	**[−0.02; 0.16]**	—	**100.0%**
Heterogeneity: $I^2 = 64\%$, $p < 0.01$								

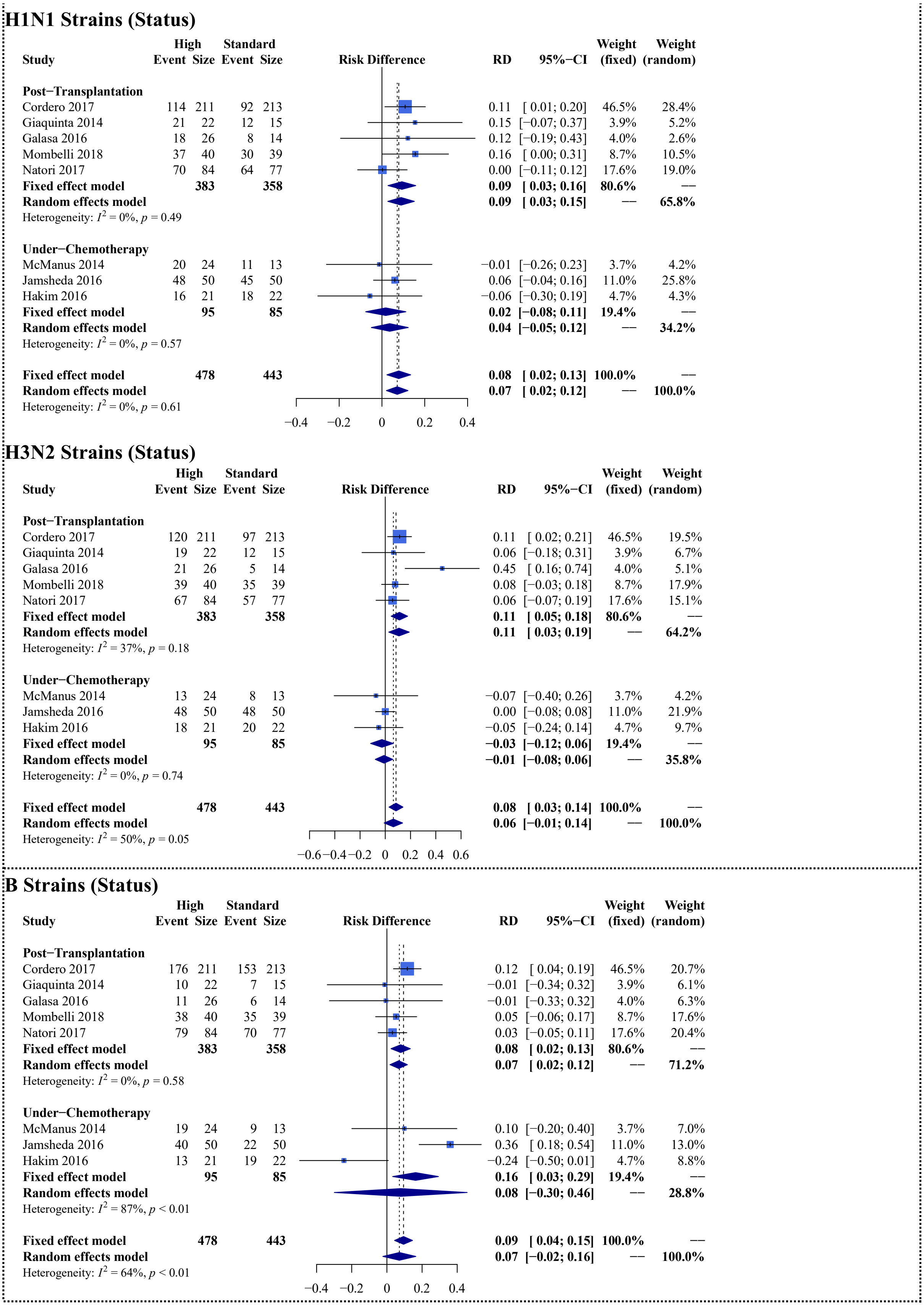

Figure S3. Forest plots of subgroup analyses with variables of Dosage, Mean Age and Status in SeroProtection Outcomes.

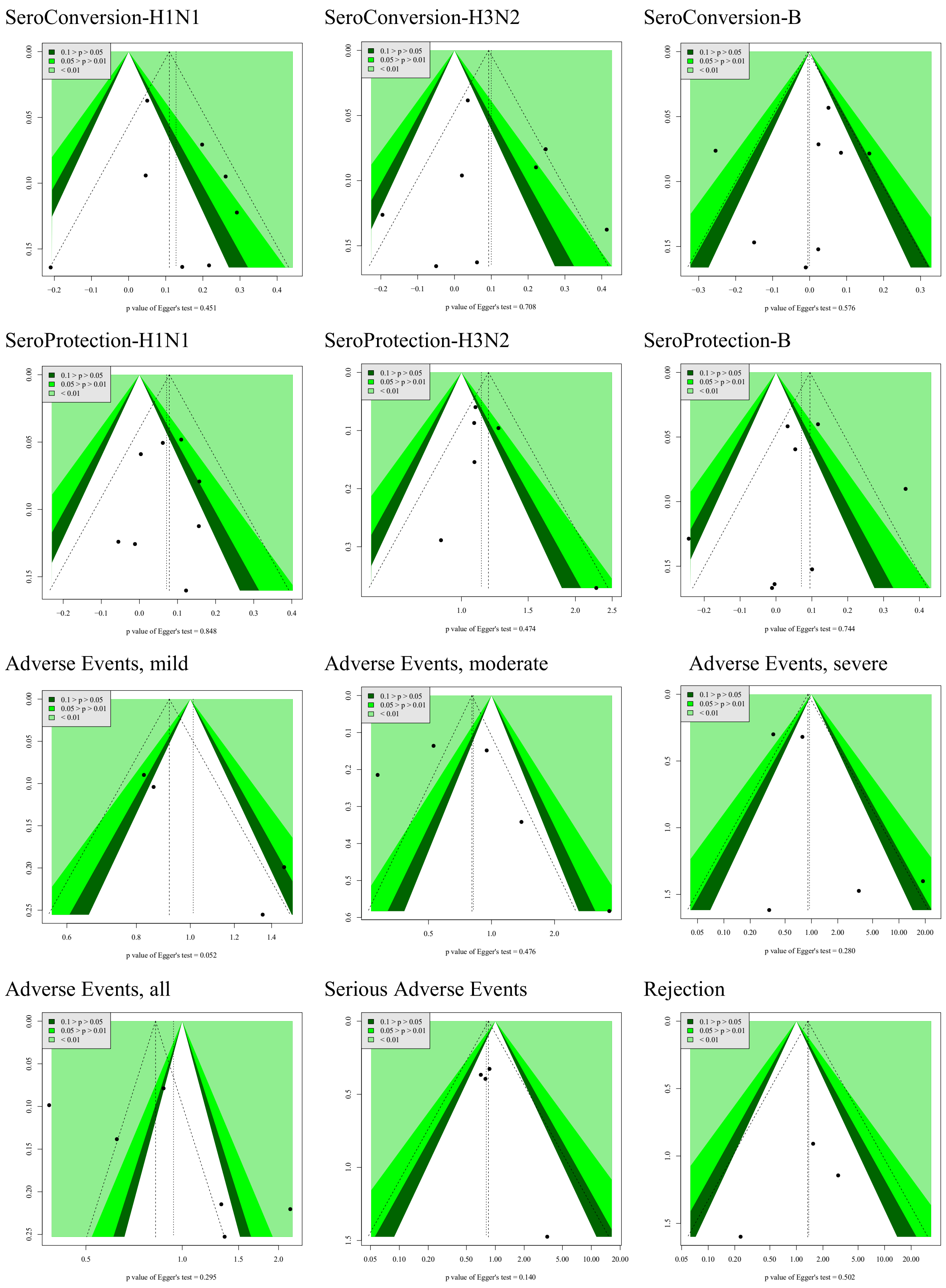

Figure S4. Funnel plots of major outcomes in participants with diabetes for aspirin intervention trials.
